# DMCNN: A Deep Multiscale Convolutional Neural Network Model for Medical Image Segmentation

**DOI:** 10.1155/2019/8597606

**Published:** 2019-12-26

**Authors:** Lin Teng, Hang Li, Shahid Karim

**Affiliations:** ^1^Software College, Shenyang Normal University, Shenyang 110034, China; ^2^Institute of Image and Information Technology, Harbin Institute of Technology, Harbin 150000, China

## Abstract

Medical image segmentation is one of the hot issues in the related area of image processing. Precise segmentation for medical images is a vital guarantee for follow-up treatment. At present, however, low gray contrast and blurred tissue boundaries are common in medical images, and the segmentation accuracy of medical images cannot be effectively improved. Especially, deep learning methods need more training samples, which lead to time-consuming process. Therefore, we propose a novelty model for medical image segmentation based on deep multiscale convolutional neural network (CNN) in this article. First, we extract the region of interest from the raw medical images. Then, data augmentation is operated to acquire more training datasets. Our proposed method contains three models: encoder, U-net, and decoder. Encoder is mainly responsible for feature extraction of 2D image slice. The U-net cascades the features of each block of the encoder with those obtained by deconvolution in the decoder under different scales. The decoding is mainly responsible for the upsampling of the feature graph after feature extraction of each group. Simulation results show that the new method can boost the segmentation accuracy. And, it has strong robustness compared with other segmentation methods.

## 1. Introduction

Medical imaging makes a critical difference in clinical diagnosis [1–3]. Recently, with the progress of medical imaging technology and the continuous development of artificial intelligence image processing, medical image processing technology has gradually developed into a key research field. It is vital in clinical application. The aim of medical image segmentation technology is to segment the interested part by some deep automatic segmentation algorithms and make the segmentation results as close as possible to the original structure of the region [4]. Segmentation of medical image has big significance in clinical diagnosis and pathological diagnosis. Measuring lesion volume with segmented images can assist doctors to determine the disease and make treatment plans [5].

Medical imaging segmentation (MIS) is an indispensable stage in ROI (region of interest) extraction, quantitative analysis, and 2D reconstruction. The images will be segmented with the same or similar features (such as intensity, color, and texture) into separated areas, particularly to extract the lesion areas with special meanings or other regions of interest (ROI) from the complex background, so as to provide basis for clinical analysis [6]. Magnetic Resonance Imaging (MRI) uses the principle of nuclear magnetic resonance, which not only has high soft tissue resolution but also provides rich and high-resolution three-dimensional brain tissue information. Therefore, how to segment the medical images accurately in MRI images is becoming a challenging task in medical image research [7].

Through the analysis of research status, we summarize three kinds of traditional MIS methods: (1) manual segmentation method, which is tedious, excessive labor, subjective, prone to error, and not suitable for large-scale research [8]; (2) semiautomatic segmentation method, which requires accurate control of prior parameters and consumes much time in the process of parameter tuning [9]; (3) the traditional segmentation methods such as graph-based, deformation model, and active appearance model [10–12], which is based on simple registration method. However, due to the differences between the hippocampus, in terms of the segmentation efficiency and accuracy, a simple registration method is still not ideal.

Currently, deep learning has attracted more attention, and the model based on deep CNN and its variants have been diffusely used in various fields of medical image processing and also achieved better results [13–15]. For example, Cha [16] presented MR brain image automatic segmentation method using a CNN network. The method was independent on explicit features only requiring a single MR (magnetic resonance) image. Lu et al. [17] used two-dimensional convolutional neural network to evaluate the segmentation of electron microscope images. Zhang et al. [18] adopted deep CNN to evaluate the segmentation of multimodal brain images. Although the models based on CNN have obtained better performance, these methods have a common problem, namely, all networks take image block as input, due to a large amount of overlapping image blocks, the redundant computation will increase the time cost for testing the network, and the image block size will influence the capability of the trained network. To solve the problem of image segmentation, many researchers have come up with many approaches based on the fully convolutional network (FCN) model to remedy limitations of image segmentation. FCN can take the entire image as the input of the network and generate the corresponding output of the whole image, thus avoiding the problems caused by the use of image blocks. However, it had low efficiency. So, a deep multiscale CNN model is put forward for medical image segmentation. The major contributions are illustrated as follows:

1. First, we extract the regions of interest from the raw medical images. Then, the data augmentation is operated to obtain more training dataset. The encoder, U-net, and decoder models are used for constructing our proposed segment framework.
2. Encoder is mainly responsible for feature extraction of 2D image slice.
3. In different scales of the decoder, it will acquire the features of each encoder's block by deconvolution operation, and then the U-net joins them together.
4. The decoding is mainly responsible for the upsampling of the feature graph after feature extraction of each group.

This paper is originated as follows. In Section 2, related works are introduced for the image segmentation.  Section 3 describes the proposed DMCNN in detail. In Section 4, we conduct experiments and give analysis.  Section 5 concludes the work.

## 2. Related Works

### 2.1. Inception Model

In order to make the convolutional neural network have better learning ability, the most direct and effective method is to make the network layer deeper. However, there are some disadvantages in this operation: (1) if the training number and dataset are limited, more parameters will easily lead to overfitting; (2) if the network is larger, it is hard to utilize due to the greater endless computation; (3) the deeper the network is, the gradient will disappear, which leads to the diffusion of gradient. Under this situation, it is difficult to optimize the network model. Inception v1 is proposed in 2014 [19], the convolution layer of 1 × 1, 3 × 3, and 5 × 5, and pooling layer of 3 × 3 are stacked together, which increases the width of the network and also enhances the adaptability of the network in terms of the scale. This operation can extract features from different scales. An important improvement in Inception v31 is decomposition, the two-dimensional convolution of *N* × *N* is divided into two-dimensional convolutions of 1 × N and *N* × 1. The advantage of this method is that it can not only accelerate the computation but also increase the nonlinearity of the network.

### 2.2. Batch Normalization

BN aims to add a standardized processing for the input data of each layer in the training process of neural network, which also belongs to the network layer. Previously, we mentioned that in addition to the output layer of the network, the parameters of the lower layer of the network are updated during the training, which caused the change of the distribution of the input data in the latter layer. In each layer, it is better to add a preprocessing operation. For example, the data in the third layer of network are normalized. Then, it inputs the third layer for calculation, so that we can solve the problem of “Internal Covariate Shift” [20–22]. By introducing batch standardization method, the network's convergence speed will be greatly increased. The overfitting can also be controlled. The dropout and regularization operation will be realized with little utilization.

### 2.3. End-To-End Models and Jump Connections

Compared to the traditional image block-based convolutional neural network model, the end-to-end model utilizes the entire image as input. The entire image will be generated as output [23, 24]. The image block-based model needs to foretell each size of pixel in the slice separately. Therefore, the end-to-end model adopted in this paper can evidently decrease the time consumption when segmenting images. Generally, end-to-end models primarily include fully CNN and faster CNN. It combines the feature maps of different levels. Unlike FCN, the U-net model adopts jump connection to join the feature information obtained from the shrinking coding path and the deconvolution operation in the expanded path together which is favourable for obtaining multiscale feature information to strengthen the network's feature extraction ability.

## 3. Proposed DMCNN

### 3.1. Data Preprocessing

**Figure 1:**
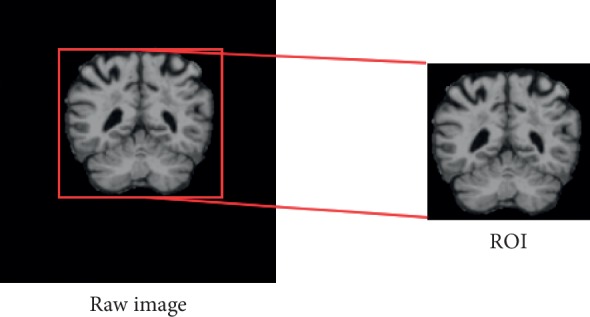
Extracted ROI.

For the acquired medical images, the region of interest should be extracted and preprocessed to serve as the samples for training and testing the network. After that, gray level regularization is carried out on all acquired ROIs. And, the mean and SD (standard deviation) are measured. The gray level regularization is assessed by subtracting mean value and dividing by SD. In the subsequent training and testing process, the extracted images are transported into the network model as samples, and the extracted region is shown in Figure 1. For example, the size of the raw image is 256 × 256, and after ROI extraction, we get the 128 × 128 patch and input it into network for training.

### 3.2. Data Extension

**Figure 2:**
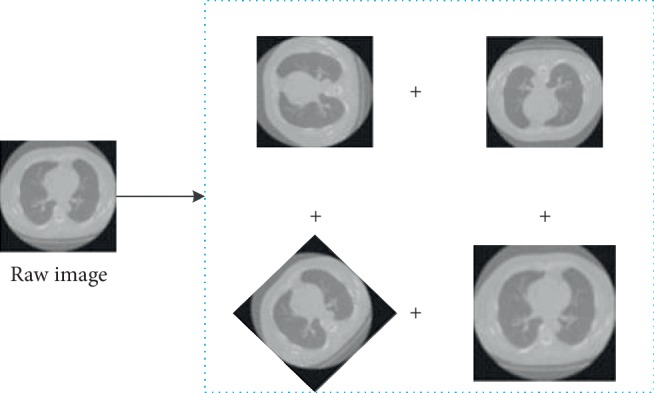
Proposed medical image segment model.

Since labeled public medical image datasets are little online and they are inconvenient to use, training a deep CNN model is troublesome [25]. In our proposed model, we first adopt some newest data argumentation methods to expand the raw data in order to increase the number of available training samples. In this paper, five data expansion methods are adopted including vertical direction reversal, random angle rotation, random translation, horizontal direction reversal, and image deformation.  Figure 2 shows an example of the image expansion.

### 3.3. Proposed Network Model

**Figure 3:**
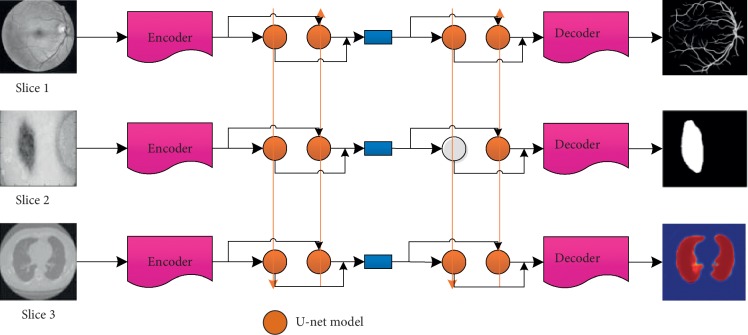
Proposed medical image segment model.

To raise the accuracy of medical image segmentation, spatial information of images and relevant information between 2D slices are effectively utilized. The proposed deep multiscale convolutional neural network model contains three parts: encoder, U-net, and decoder as shown in Figure 3.

#### 3.3.1. Encoder

**Figure 4:**
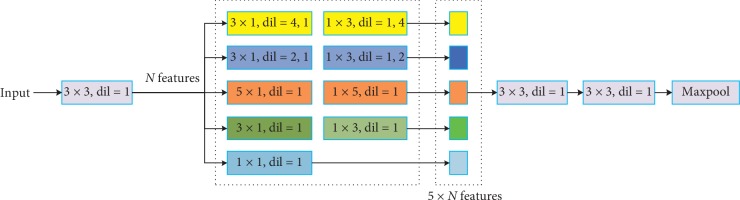
Encoder structure.

It is mainly responsible for feature extraction of 2D slices, whose network structure is shown in Figure 4. Small convolution kernel in convolutional network is conducive to capturing local information, while large convolution kernel is conducive to capturing global information. However, ROIs are different in 2D slices, and it is difficult to select an accurate and universal convolution kernel. For this purpose, we use three different convolution layers (1 × 1, 3 × 3, 5 × 5) to extract information of multiple scales in Inception V1, which can extract more features. Additionally, to reduce the amount of computation, asymmetric convolution kernel is used in this experiment to decompose the *N* × *N* two-dimensional convolution into two one-dimensional convolutions with 1 × *N* and *N* × 1.

Meanwhile, to expand the receptive field of convolution and perfectly obtain multiscale information without increasing the size of parameters, this paper adds dilated convolutions with expansion coefficients of 2 and 4, respectively. For an ordinary convolution layer of 3 × 3, the receptive field of its convolution kernel is 3 × 3. In this paper, after the employment of dilated convolution (DC), the size of parameters remains unchanged, but the receptive field of the convolution kernel becomes 7 × 7 and 15 × 15. It can be seen that DC greatly increases the receptive field of the convolution layer without increasing parameters number. As shown in Figure 4, there are three kinds of dilated convolution with expansion coefficients of 1, 2, and 4, respectively, in the encoded part, and the receptive fields of the corresponding convolution kernel are 3 × 3, 7 × 7 and 15 × 15, respectively. After using the three receptive fields with different sizes, it is not only conducive to feature extraction but also conducive to better capturing the pathological area features.

Then, after cascading the feature graphs extracted from five different convolution layers, we utilize two ordinary 3 × 3 convolution layers in feature extraction process. Finally, to reduce the size of the feature map, it connects a maximum pooling layer. In Figure 4, the number of channels in each convolution layer is 16. Considering the convergence of networks, batch normalization is added behind the convolution layer, and ReLu layer is used as the activation function.

#### 3.3.2. U-Net Model

**Figure 5:**
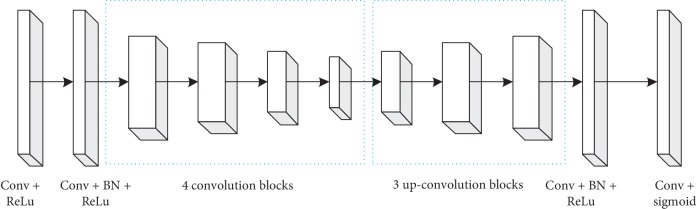
Deep multiscale U-net model.

Figure 5 shows the proposed U-net network structure employed. The U-net network structure contains two parts as follows:

- Contractile encoder part on the left. It processes input medical images.
- Decoder part on the right. It produces labeled output.
- The skip connection. It can cascade the features of each block in the encoder. Features are obtained by deconvolution operation in the decoder.

The U-net model in this paper is displayed as Figure 5.

The proposed entire U-net network consists of twenty-eight convolution layers. In here, twenty-four convolution layers are spread over four convolution blocks and four deconvolution blocks. The contraction encoder in deep multiscale U-net covers four convolution blocks shown in left of Figure 4. Each convolutional block contains two convolutional layers (conv). Each convolutional layer utilizes a 3 × 3 convolutional kernel to carry out the convolution. The step size is 1. Synchronously, each convolutional layer follows a BN layer and a ReLu layer to modify the network performance. ReLu activation function has sparse ability, and it can better learn the relatively sparse features from the effective data dimension and play the role of feature automatic decoupling. In each convolution block, its first convolution layer can double feature graphs. The number of feature graphs will be increased to 64. After four convolution layers, the number of feature graphs is increased from 64 to 1024. Between each convolution block, the sampling method of traditional U-net adopts maximum pooling. In this paper, we use the 2 × 2 convolution kernel with step length 2 for down-conv operation to achieve a convolution block feature on the image of the sampling operation. Through this down-conv operation, the size of feature graph is reduced by half with iterative deepening, so that the size of input original image is decreased from 128 × 128 layer by layer to 8 × 8.

**Figure 6:**
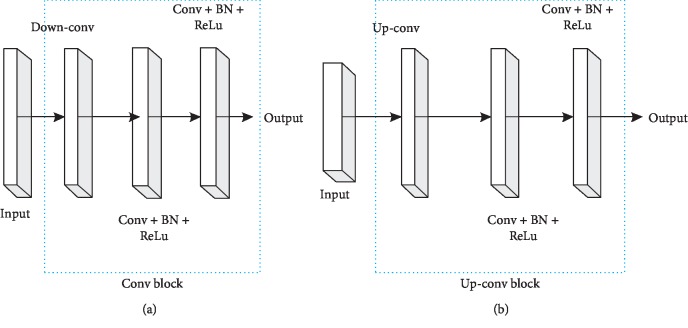
Convolution block and Up-convolution block structure.

The expansion decoder of the U-net model contains three deconvolution blocks as shown in the right of Figure 6. The deconvolution up-conv operation adopts the 3 × 3 convolution kernel. The step size is 2, and the size of the feature graph is increased by twice the original size through the deconvolution operation. This process can recover the feature graph as the raw input image in the last deconvolution block. Meanwhile, the number of feature graphs is halved after each deconvolution operation. The feature graphs obtained by deconvolution are cascaded with the corresponding feature graphs in the convolution block as the feature input of the deconvolution block. Two convolution layers are in each deconvolution block. The 3 × 3 convolution kernel is utilized (the step size is 1). The first convolution layer will reduce the number of feature graphs by half after every cascading. Not exactly the same as the original U-net structure, the presented U-net structure is filled with zero filler in each convolution layer. According to formula (1), the output size of the deep multiscale CNN model can be guaranteed to be consistent with the input image data size by using zero padding:

(1) $$\begin{array}{c} I_{\text{output}}=\frac{\left(I_{\text{input}}-F+2P\right)}{S+1}, \end{array}$$

where *I*_input_ and *I*_output_ are on behalf of the input and output images' size in DMCNN, respectively. *F* represents the convolution kernel with size 3 × 3. *P* denotes the fill size with 1 × 1. *S* = 1 stands for step size in this paper.

At the end of the proposed U-net model in this paper, we skillfully adopt a convolution layer (whose size is 1 × 1) to lessen the number of feature graphs to 1. The final output will be disposed by the Sigmoid function. We can obtain the value of each pixel between 0 and 1. The lesion area is a probability distribution. Through the above processing, the final image is considered as the probability graph of DMCNN. The value corresponding to each pixel indicates the probability that the point belongs to the lesion.

#### 3.3.3. Decoding Part

**Figure 7:**
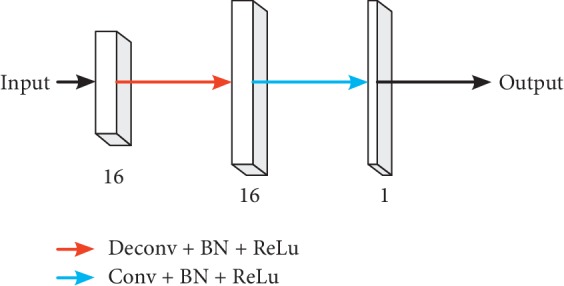
Decoder structure.

It is mainly responsible for upsampling the feature graph after extracting each group feature, and the structure is shown in Figure 7. The decoder section contains one deconvolution layer and one convolution layer. Both deconvolution and convolution have batch-normalization and ReLu. After the upsampling, the feature graph becomes the same resolution as the input image. Finally, the final segmentation result is obtained by softmax classifier to analyze the end-to-end segmentation.

### 3.4. Loss Function

Different from the commonly used pixel point-based softmax loss function [26, 27], Dice loss function is based on region loss function. In medical image segmentation, Dice index is often used to measure the overlap rate between the object and the detection area. If the Dice value is larger, then the overlap degree is higher, and the segmentation effect is better. However, Dice index cannot be directly used as a loss function, so we use the improved Dice function. Dice function is a function that gives feedback to network parameters after independent evaluation for each area [28]. The calculation form and process of Dice function are in good agreement with medical image segmentation. Therefore, Dice loss function used in this paper is defined as follows:

(2) $$\begin{array}{c} \text{Dice}\left(g,p\right)=1-\frac{2\sum_{i}^{v}p_{i}g_{i}}{\sum_{i}^{v}\left(p_{i}^{2}+g_{i}^{2}\right)}, \end{array}$$

where *g* stands for the ground truth. *p* is the predicted value. *v* is the number of pixels in each image block. Dice always is used as a loss function, when comparing the probability graph with the labeled. The background part whose labeled value is 0 will not be calculated into the loss to avoid the situation of unbalanced category and accelerate the convergence of the network and improve the segmentation accuracy.

## 4. Results and Discussion

### 4.1. Dataset and Evaluation Index

The dataset is from ADNI (Alzheimer's Disease Neuroimaging Initiative: adni.loni.usc.edu) [29, 30]. In this advanced researches, 100 groups of brain MRI images and segmented hippocampal tags are obtained from ADNI library. From this group, 80 groups are randomly selected for cross-validation, and the remaining 20 groups are for testing.

To improve the segmentation veracity, this study preprocesses the data with three steps. First, consider that the hippocampus only accounts for a small part of the whole brain MRI image and other parts are invalid areas. The pixel values in brain MRI are statistically analyzed, and the images are cropped into 80 × 80 × 40 including the hippocampus and the blank area around it. In this way, invalid background information can be reduced without any influence on the integrity of valid information. Second, to accelerate the convergence of the network and consider the inconsistency of the pixel values of MRI images in ADNI, the mean and SD methods are utilized to normalize the images. Thirdly, making allowances for the small number of samples in the dataset, we enhance the obtained MRI images by left rotation and right rotation and finally obtain 400 MRI images.

To accurately reflect the performance differences between algorithms, we use uniform platform. The hardware environment of the experiment is NVIDIA GTX1060Ti, Intel Corei7 processor, and the software environment was Keras2.2.4. In the experiment, glorot normal distribution method is used to initialize the weight. The image size is 300 × 300 pixel used in this section. Execution environment is GPU and Geforce GTX 1060. The parameters used in DMCNN are given in Table 1.

To quantitatively evaluate the performance of the new approach, dice similarity coefficient (DSC), sensitivity (SEN), and predictive positivity value (PPV) are selected as the evaluation indexes for the medical image segmentation result. They are defined as follows:

(3) $$\begin{array}{c} \text{DSC}=2\frac{P\cap T}{P+T},\\ \text{SEN}=\frac{P\cap T}{T},\\ \text{PPV}=\frac{P\cap T}{P}, \end{array}$$

where *P* denotes the lesion region segmented by the presented algorithm. *T* expresses the region of Ground truth. *P*∩*T* represents the pixel region of the intersection between the algorithm's segmentation region and the true segmentation region.

### 4.2. Comparative Analysis of Segmentation

In this paper, 100 images before augmentation and 400 images after augmentation are segmented by the proposed method. The evaluation indexes in above section are used for evaluation. The comparison results of DSC, SEN, and PPV are given in Table 2.

From Table 2, it reveals that data augmentation can greatly improve the segmentation accuracy. This also fully proves the importance of datasets in the deep learning model construction process. The size of the datasets can directly affect the learning capability of the model.

To verify that DMCNN can effectively capture information between slices, we conduct the comparison between multiscale convolutional neural network and single-scale CNN in this paper. Other conditions remain unchanged. The effectiveness of the proposed model can be observed from Table 3.

We can see that the segmentation accuracy of DMCNN is significantly higher than that of single-scale CNN, which further verifies that DMCNN can better learn more feature information between slice sequences than single-scale CNN.

Meanwhile, we study the effect of different network models on experiment results. Two representative segmentation methods including U-net and 2D U-net network model are compared with our deep multiscale U-net given in Table 4. The segmentation accuracy obtained by the DMCNN method is higher than that of the other two methods, indicating that it can extract features more efficiently and improve the segmentation accuracy.

Compared with the multiple groups of up- and downsampling layers in U-net and 2D U-net networks, the proposed network model only contains one up- and downsampling layer, which greatly reduces the size of the parameters. The number of parameters of the encoding part and decoding part is below 5000, which significantly reduces the computation time in this article.

We also conduct comparison experiment with state-of-the-art segment methods including TLWK [31], MNF [32], and SUSAN [33] on our medical data. The results are given in Table 5.

TLWK adopted the traditional random forest regression method, and MNF adopted the multiscale method. They are all automatic segmentation methods, due to the large gap between different individuals, and the accuracy and efficiency of segmentation are often not ideal, which is not as high as the precision of the automatic segmentation method in this paper. SUSAN simply improves 2D U-net, so the results are not very good. In general, the proposed method combining convolution neural network and multiscale U-net model is superior to other current methods for medical image segmentation. And, the time consumption is shorter than other methods too.

**Figure 8:**
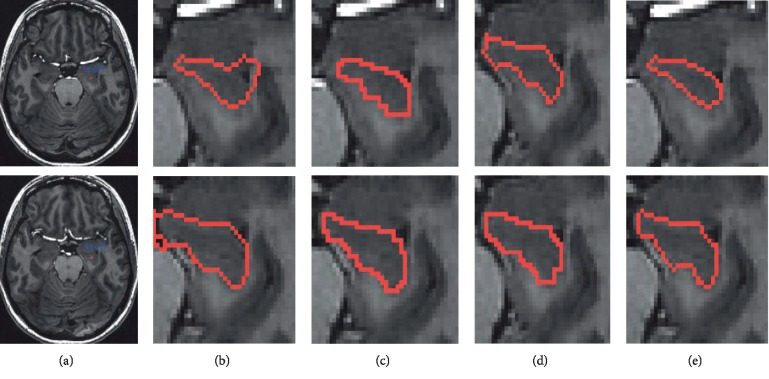
Segmentation of hippocampus with different algorithms. (a) Original images of hippocampus with initial seed points (blue). Results of the (b) TLWK model; (c) MNF method; (d) SUSAN method; (e) DMCNN method.

**Figure 9:**
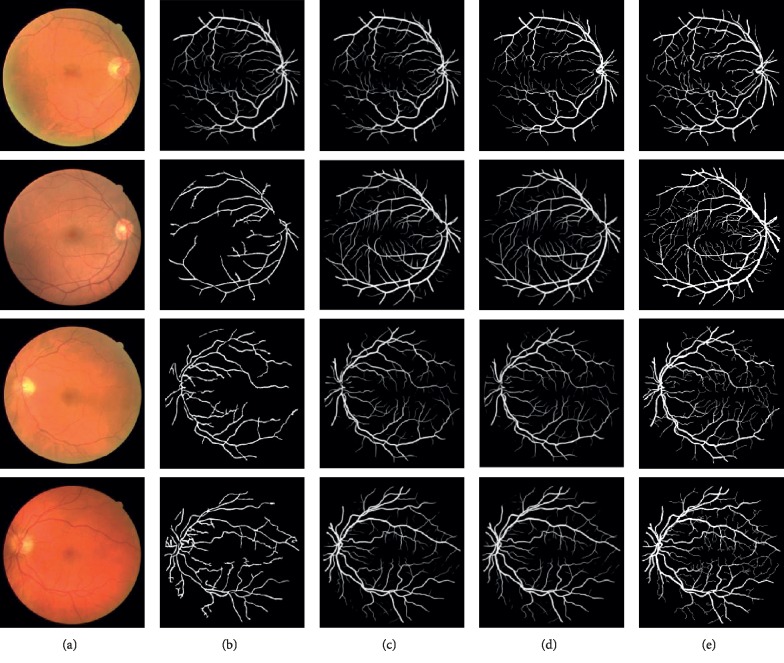
Segmentation results: (a) original image; (b) TLWK method; (c) MNF method; (d) SUSAN method; (e) DMCNN method.

**Figure 10:**
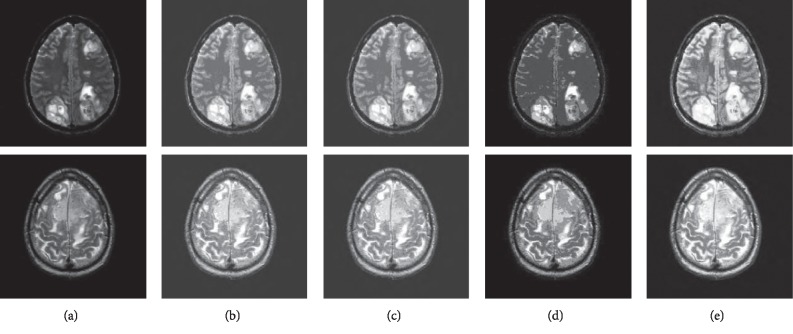
Segmentation results: (a) original image; (b) TLWK method; (c) MNF method; (d) SUSAN method; (e) DMCNN method. The first row is sarcoma, and the second is meningioma.

Figures 8–10 are the segmentation comparison results in terms of hippocampus, retinal blood vessel, sarcoma, and meningioma.

Given the segmented image, the IoU measure gives the similarity between the predicted region and the ground truth region for an object and is defined by following equation:

(4) $$\begin{array}{c} \text{IoU}=\frac{\text{TP}}{\text{FP}+\text{TP}+\text{FN}}, \end{array}$$

where TP, FP, and FN denote the true positive, false positive, and false negative counts, respectively. The results are given in Table 6. We can see that the proposed segment method has the better result.

## 5. Conclusions

This paper proposes a medical image segmentation method based on multiscale convolutional neural network. This method can realize automatic segmentation of medical images and has high accuracy of segmentation. The CNN model in this paper not only reduces the amount of computation but also effectively captures multiscale information. In addition, the use of U-net fully mines the relevant information between slice sequences. Taking relevant medical image segmentation as an example, the experimental results on ADNI database show that the segmentation method in this paper is superior to other methods. The proposed method can perform segmentation tasks more easily and accurately. In the future, studying on deeply deep learning methods to segment images and applying them into different types of images and different practical engineering are warranted.

## Figures and Tables

**Table 1:** Parameters in this experiment.

Learning rate	0.001
Batch size	8
L2 regularization	0.0001

**Table 2:** *Hippocampus* segment results with DMCNN.

Images	DSC	SEN	PPV
100	88.37	89.65	89.54
400	**90.58**	**91.92**	**92.73**

**Table 3:** *Hippocampus* segment results with DMCNN and single-scale CNN.

Method	DSC	SEN	PPV
Single-scale CNN	86.54	86.95	87.31
Multiscale CNN	**91.26**	**90.89**	**91.57**

**Table 4:** *Hippocampus* segment results with different U-net models.

Method	DSC	SEN	PPV
U-net	89.26	88.73	89.45
2D U-net	89.65	89.21	90.14
Proposed	**91.23**	**90.87**	**91.58**

**Table 5:** *Hippocampus* segment results with different methods.

Method	DSC	SEN	PPV	Time
TLWK	84.62	83.17	86.54	11.5 s
MNF	87.13	86.45	87.53	11.8 s
SUSAN	88.62	88.92	89.85	10.2 s
DMCNN	**92.54**	**91.87**	**92.08**	**8.5** **s**

**Table 6:** IoU results with different methods.

Method	*Hippocampus*	Retina	Sarcoma	Meningioma
TLWK	72.48	81.73	75.48	73.69
MNF	78.31	84.56	79.35	78.24
SUSAN	82.37	88.57	82.54	81.53
DMCNN	**89.62**	**90.24**	**89.66**	**88.74**

## Data Availability

The data used to support the findings of this study are available from the corresponding author upon request.
